# Combined Bioinformatic Analysis and RNA Sequencing Uncover Key Tinnitus-Related Molecules in the Cingulate Gyrus

**DOI:** 10.3390/ijms27198543

**Published:** 2026-09-24

**Authors:** Yuan Jin, Xinyu Shi, Qiong Zhang, Bei Guo, Ying Xiong, Yao Hu, Haixia He, Pei Gao, Wei Chen

**Affiliations:** 1 Department of Otorhinolaryngology, The Central Hospital of Wuhan, Tongji Medical College, Huazhong University of Science and Technology, Wuhan 430014, China; m201975754@alumni.hust.edu.cn (Y.J.);; 2 Department of Otorhinolaryngology, Union Hospital, Tongji Medical College, Huazhong University of Science and Technology, Wuhan 430022, China; sxy2017@hust.edu.cn

**Keywords:** cingulate gyrus, tinnitus, *AKT1*, bioinformatic analysis, RNA sequencing

## Abstract

Tinnitus arises from maladaptive plasticity and abnormal synaptic function, not only in auditory pathways but also in limbic regions. Among these, the cingulate gyrus plays a pivotal part in shaping the emotional burden and sensory filtering disturbances often seen in tinnitus patients. Yet, the specific molecular players operating within this region remain poorly understood, and most existing bioinformatic predictions have not been tested in living systems. Here, we combined multi-database mining with in vivo transcriptomic profiling to identify and validate key proteins and pathways linked to tinnitus in the cingulate gyrus. We built and compared protein–protein interaction networks representing auditory perception, acoustic challenge, and tinnitus states, thereby pinning down candidate hub molecules. To verify these candidates, we used a noise-induced mouse model of tinnitus, confirmed the phenotype via gap prepulse inhibition of acoustic startle (GPIAS), and then performed RNA-seq and qPCR on cingulate gyrus tissue. The bioinformatic screens pointed to *AKT1* and *TNF* as central hubs, suggestive of an inflammatory–survival signaling axis. In our animal experiments, RNA-seq showed a clear rise in *Akt1* transcript levels in the tinnitus group, and functional enrichment analysis pointed to marked changes in extracellular matrix organization, cell adhesion, and cytokine receptor pathways. qPCR further corroborated the *Akt1* upregulation. Taken together, our integrative strategy establishes *AKT1* and *TNF* as topologically central hub proteins in the cingulate gyrus tinnitus network, and validates *Akt1* upregulation in vivo, offering potential biomarker candidates and therapeutic leads for future studies.

## 1. Introduction

Tinnitus is defined as the conscious perception of an auditory sensation in the absence of a corresponding external stimulus [1]. The global prevalence of tinnitus among adults is estimated at 10% to 30% [2,3,4,5,6], and approximately 15% of individuals will experience persistent or long-term tinnitus during their lifetime [3]. Tinnitus exhibits marked variability in clinical impact: while many patients are minimally affected, others experience severe, life-disrupting symptoms, including cognitive impairment, emotional distress, sleep disturbances, and reduced quality of life [7]. Tinnitus may be constant or intermittent, localized to the ears or head, and typically has an insidious onset, with intensity often exacerbated by stress [8]. This profound phenotypic heterogeneity has been a major barrier to advancing tinnitus research and treatment [9].

Recent research indicates that tinnitus primarily arises from maladaptive neuroplasticity within central auditory pathways rather than abnormal hyperactivity in the damaged cochlea or auditory nerve [10]. Hearing loss, especially hidden cochlear synaptopathy, triggers reduced afferent input and induces enhanced spontaneous firing, increased neural synchrony, and central gain amplification in the central auditory system [11]. However, hearing loss alone is insufficient to cause tinnitus, as most individuals with hearing impairment do not develop tinnitus, suggesting that additional modulatory factors are required for the manifestation of tinnitus [12]. Emerging studies have demonstrated that emotional stress, emotional exhaustion, and long-term stress serve as critical secondary factors that promote the transition from hearing deprivation to clinical tinnitus [13,14]. Approximately half of tinnitus patients report exacerbated symptoms under psychological stress, and unstable emotional states are closely associated with increased tinnitus loudness and distress [15]. Functional brain imaging and electroencephalography studies further demonstrate aberrant connectivity between limbic emotional regions and auditory networks in tinnitus [16,17,18]. Specifically, the subgenual, rostral, and dorsal anterior cingulate gyrus are critically involved in tinnitus-related distress [17,19]. Tinnitus-related distress is closely associated with alpha and beta oscillations in the rostral–dorsal anterior cingulate gyrus, which acts as a key node in a network including the amygdala, anterior cingulate gyrus, insula, and parahippocampus [17,19].

The pregenual anterior cingulate gyrus has been identified as a critical top-down inhibitory gatekeeper that modulates sensory information flow between the thalamus and auditory cortex via the thalamic reticular nucleus [16]. As a core region of the corticolimbic circuit, the anterior cingulate cortex undergoes maladaptive plasticity under chronic stress, accompanied by neurochemical imbalance and reversible structural atrophy [20]. Although distress is recognized as a major comorbidity of tinnitus, the molecular mechanisms underlying the cingulate gyrus-mediated interaction between distress and tinnitus remain largely unclear. In particular, bioinformatic signatures of protein–protein interaction (PPI) networks and key molecules associated with cingulate gyrus-related tinnitus pathogenesis have not been systematically investigated. These pathological processes of tinnitus rely on extensive and dynamic protein–protein interactions, and bioinformatics approaches represent a powerful strategy to identify core functional proteins. By quantitatively analyzing and comparing interaction networks under physiological and tinnitus-related conditions, key proteins and potential biomarkers can be effectively identified to clarify the underlying molecular mechanisms. Nevertheless, most existing bioinformatic studies on tinnitus rely on single-source data mining, focus predominantly on classical auditory structures such as the auditory cortex and inferior colliculus, and generally lack corroboration from in vivo experimental evidence.

To tackle these issues, we designed a two-stage strategy that first screens for candidate molecules through multi-database bioinformatic analysis, and then validates the findings in vivo using transcriptomic approaches. Specifically, we built protein–protein interaction networks for auditory perception, acoustic stimulation, and tinnitus, drawing on publicly available resources such as GeneCards, STRING, and DAVID. By comparing these networks, we identified hub proteins with high topological importance and their associated functional pathways. For the experimental leg, we chose a well-established noise-induced mouse model, which closely mirrors the most common cause of acquired tinnitus in humans. We confirmed the tinnitus phenotype using the gap prepulse inhibition of acoustic startle (GPIAS) test, and then collected cingulate gyrus tissues for RNA-seq and qPCR to cross-check the bioinformatic predictions and to uncover any novel pathological signatures. Our analysis pointed to *AKT1* and *TNF* as the central hub proteins in the tinnitus network. The subsequent in vivo experiments not only verified a marked upregulation of *Akt1* in the cingulate gyrus of tinnitus mice, but also revealed that extracellular matrix remodeling may be an unrecognized feature of the condition. Overall, this work clarifies the molecular landscape of the cingulate gyrus in tinnitus and offers promising biomarker candidates and therapeutic leads for future mechanistic and translational studies.

## 2. Results

### 2.1. Gene Lists of Auditory Perception, Acoustic Stimulation, and Tinnitus

The gene lists for auditory perception (AP, n = 82), acoustic stimulation (AS, n = 54), and the tinnitus (Tin, n = 104) process differ in both the types and numbers of genes. The Venn diagram (Figure 1) shows a remarkable overlap of genes associated with the AP, AS, and the Tin process (Figure 1 and Table 1). A total of 156 unique elements were identified.

Notably, the 48 genes uniquely enriched in the Tin group included core hub candidate *AKT1*, *TNF*, *BCL2*, and dozens of extracellular matrix (ECM) regulatory transcripts (*FLNB*, *PTK2*, *CFAP410*), preliminarily indicating that inflammatory–survival signaling and ECM remodeling may participate in tinnitus pathological processes.

### 2.2. GO Enrichment Analysis

Gene ontology (GO) enrichment analysis was used to characterize the three gene lists and assign related genes to cellular components (CCs) and biological processes (BPs). The GO enrichment analysis indicated 25 chart records for the AP group, 25 chart records for the AS group, and 125 records for the Tin group (*p* < 0.01). The top five GO terms with the highest significance for cellular components (CCs) and for biological processes (BPs) are shown in Table 2.

The top five GO-CC terms of the AP, AS, and Tin lists reflect core neural structures in the cingulate gyrus that underlie auditory processing and synaptic regulation, including synapse, glutamatergic synapse, axon, dendrite, and cytosol (Table 2). The top five GO-BP terms of the three groups indicate that processes related to synaptic transmission and neural activity regulation are dominant across all conditions. In the AP group, representative biological processes include “response to auditory stimulus”, “detection of mechanical stimulus involved in sensory perception of sound”, and “regulation of synaptic vesicle exocytosis”, consistent with normal auditory perception and physiological synaptic function. In the AS group, GO-BP terms such as “chemical synaptic transmission”, “G protein-coupled receptor signaling pathway coupled to cyclic nucleotide second messenger”, and “fear response” are enriched, indicating an altered activity state of the auditory system under stimulation. In the Tin group, unique and significant GO-BP terms comprise “positive regulation of *ERK1* and *ERK2* cascade”, “regulation of gene expression”, “positive regulation of action potential”, and “intrinsic apoptotic signaling pathway in response to DNA damage”, indicating pronounced changes in synaptic plasticity, neuronal hyperexcitability, and cellular homeostasis in the cingulate gyrus during tinnitus. These results indicate that genes in the Tin group are more related to intracellular signaling cascades, transcriptional regulation, neuronal excitability, and apoptotic processes in the cingulate cortex. Genes activated during tinnitus drive abnormal intracellular signaling and extracellular microenvironment remodeling, rather than basic physiological auditory function. Genes associated with AP and AS show apparent association with basic synaptic structure and physiological synaptic transmission. However, these enriched synaptic-related GO terms partly originate from the shared “synaptic transmission” keyword used for generating all three GeneCards-derived gene lists, and may reflect the general synaptic molecular background within the cingulate gyrus under auditory-related states, rather than demonstrating specific functional enrichment unique to AP or AS gene sets.

### 2.3. PPI Network of the AP, AS, and Tin Processes

To characterize protein interaction patterns across physiological and pathological auditory states, PPI networks were separately constructed for AP, AS, and Tin gene lists, and topological parameters of each network were quantitatively compared (Figure 2, Table S1). Node and edge numbers, average neighbor count, network heterogeneity and centralization increased progressively from AP to AS to Tin, demonstrating that the Tin network is substantially more complex than the AP and AS networks. Frequency distribution analysis of node degree and combined interaction score was conducted to screen high-degree proteins (HDPs) and high-score interaction proteins (HSIPs) (Figure 3, Table 3). The 90th percentile of the global CS distribution was applied as the cutoff to identify HSIPs, and the top two HDPs in each network were extracted as core regulatory nodes (Table 4). *CHAT* and *TARDBP* represented key proteins in the AP network, and *SLC6A3* and *DRD1* dominated the AS network, while *TNF* (degree = 22) and *AKT1* (degree = 21) were the two most prominent hub proteins in the Tin network, representing a putative inflammatory–survival signaling module predicted by PPI analysis. GO enrichment of these key proteins further revealed functional differentiation: AP hubs mediated cholinergic auditory transmission, AS hubs regulated dopaminergic stress signaling, and Tin hubs participated in anti-apoptosis, inflammatory response and cellular survival pathways that potentially modulate extracellular matrix homeostasis (Table 4). Taken together, multi-database bioinformatic screening identified *AKT1* and *TNF* as central hub molecules underlying tinnitus pathogenesis in the cingulate gyrus, and predicted extracellular matrix remodeling as a potential pathological feature requiring further in vivo transcriptomic verification.

**Table 3:** Key proteins in the AP, AS, and Tin networks.

HDP	Degree	Close	Betw	HSIP ^1^	Coexp	Exp	Text	CS	EB
AP									
CHAT	4	0.722	0.185	CHRNA9	0.086	0	0.792	0.801	1.23
				CHRNA10	0.086	0	0.763	0.774	1.11
TARDBP	3	0.7	0.148	FUS	0.207	0.73	0.999	0.999	2.89
				PHB2	0.06	0.163	0.522	0.591	0.45
AS									
SLC6A3	11	0.8	0.211	DRD4	0.054	0	0.96	0.96	3.12
				PRKCA	0	0.9	0.174	0.913	2.67
DRD1	7	0.75	0.126	SLC6A2	0.06	0	0.504	0.514	0.89
				OPRM1	0.139	0	0.546	0.592	1.01
Tin									
*AKT1*	21	0.833	0.245	HSP90AA1	0.049	0.883	0.996	0.999	4.56
				BCL2	0	0.9	0.934	0.993	4.41
*TNF*	22	0.84	0.261						

Abbreviations: Close—closeness centrality; Betw—betweenness centrality; HDP—high-degree protein; HSIP—high-score interaction protein; Coexp—coexpression; Exp—experimentally determined interaction; Text—automated text mining; CS—combined score; and EB—edge betweenness. ^1^: HSIPs was identified by the 90th percentile of the global CS distribution.

**Table 4:** GO processes of key proteins of the cingulate gyrus in the AP, AS, and Tin processes (*p* < 0.05).

AP (13 Chart Records)	AS (23 Chart Records)	Tin (18 Chart Records)
Cellular components: *p* = 6.72 × 10^−3^ to 8.38 × 10^−3^ Fold enrichment: 27.97–533.54	Cellular components: *p* = 4.95 × 10^−4^ to 6.19 × 10^−2^ Fold enrichment: 2.3–70.3	Cellular components: *p* = 2.18 × 10^−4^ to 5.75 × 10^−3^ Fold enrichment: 20.89–277.44
Neuron projection (CHAT, CHRNA9, CHRNA10, 3) Cholinergic synapse (CHRNA9, CHRNA10, 2) Neurotransmitter receptor complex (CHRNA9, CHRNA10, 2) Intracellular membraneless organelle (FUS, TARDBP, 2) Acetylcholine-gated channel complex (CHRNA9, CHRNA10, 2) Transmembrane transporter complex (CHRNA9, CHRNA10, 2) Cation channel complex (CHRNA9, CHRNA10, 2) Postsynaptic specialization membrane (CHRNA9, CHRNA10, 2)	Presynaptic membrane (DRD1, SLC6A2, SLC6A3, 3) Plasma membrane (PRKCA, DRD1, OPRM1, SLC6A2, SLC6A3, DRD4, 6) Cell projection (DRD1, OPRM1, SLC6A2, SLC6A3, 4) Neuron projection (OPRM1, SLC6A2, SLC6A3, 3) Axon (OPRM1, SLC6A2, SLC6A3, 3) Dendrite (DRD1, OPRM1, DRD4, 3) Membrane (PRKCA, DRD1, OPRM1, SLC6A2, SLC6A3, DRD4, 6) Synapse (DRD1, OPRM1, SLC6A2, 3) Endoplasmic reticulum (PRKCA, DRD1, OPRM1, 3) Postsynaptic membrane (DRD1, SLC6A3, 2)	Protein-containing complex (HSP90AA1, BCL2, *AKT1*, *TNF*, 4) Myelin sheath (HSP90AA1, BCL2, 2)
Biological processes: *p* = 2.82 × 10^−3^ to 8.17 × 10^−3^ Fold enrichment: 203.25–591.27	Biological processes: *p* = 1.89 × 10^−6^ to 9.95 × 10^−3^ Fold enrichment: 37.52~1084	Biological processes: *p* = 2.21 × 10^−5^ to 9.53 × 10^−3^ Fold enrichment: 14.63–867.2
Serotonin receptor signaling pathway (CHRNA9, CHRNA10, 2) Detection of mechanical stimulus involved in sensory perception of sound (CHRNA9, CHRNA10, 2) Response to auditory stimulus (CHRNA9, CHRNA10, 2) Membrane depolarization (CHRNA9, CHRNA10, 2) Amyloid fibril formation (FUS, TARDBP, 2)	Regulation of dopamine metabolic process (DRD1, SLC6A3, DRD4, 3) G protein-coupled receptor signaling pathway, coupled to cyclic nucleotide second messenger (DRD1, OPRM1, DRD4, 3) Dopamine uptake involved in synaptic transmission (SLC6A2, SLC6A3, 2) Response to xenobiotic stimulus (DRD1, SLC6A2, SLC6A3, 3) Norepinephrine uptake (SLC6A2, SLC6A3, 2) Phospholipase C-activating dopamine receptor signaling pathway (DRD1, DRD4, 2) Monoamine transport (SLC6A2, SLC6A3, 2) Behavioral response to ethanol (OPRM1, DRD4, 2) Dopamine transport (DRD1, SLC6A3, 2) Prepulse inhibition (DRD1, SLC6A3, 2) Synaptic transmission, dopaminergic (DRD1, DRD4, 2) Behavioral response to cocaine (DRD1, DRD4, 2) Regulation of transport (PRKCA, DRD4, 2) Response to amphetamine (DRD1, DRD4, 2) Response to cocaine (DRD1, SLC6A3, 2) Behavioral fear response (DRD1, DRD4, 2)	Negative regulation of extrinsic apoptotic signaling pathway in absence of ligand (BCL2, *AKT1*, *TNF*, 3) Positive regulation of nitric oxide biosynthetic process (HSP90AA1, *AKT1*, *TNF*, 3) Glucose metabolic process (*AKT1*, *TNF*, GAPDH, 3) Regulation of protein localization (HSP90AA1, BCL2, *AKT1*, 3) Response to xenobiotic stimulus (HSP90AA1, BCL2, *TNF*, 3) Regulation of apoptotic process (HSP90AA1, BCL2, *AKT1*, 3) Response to salt stress (HSP90AA1, *TNF*, 2) Nitric oxide metabolic process (HSP90AA1, *AKT1*, 2) Apoptotic mitochondrial changes (BCL2, *AKT1*, 2) Negative regulation of apoptotic process (BCL2, *AKT1*, *TNF*, 3) Positive regulation of protein localization to cell surface (*AKT1*, *TNF*, 2) Negative regulation of intrinsic apoptotic signaling pathway (BCL2, *AKT1*, 2) Negative regulation of mitotic cell cycle (BCL2, *TNF*, 2) Skeletal muscle contraction (HSP90AA1, *TNF*, 2) Negative regulation of apoptotic signaling pathway (BCL2, *TNF*, 2) Response to heat (HSP90AA1, *AKT1*, 2) Extrinsic apoptotic signaling pathway via death domain receptors (BCL2, *TNF*, 2) Apoptotic process (BCL2, *AKT1*, GAPDH, 3)

Comparative topological analysis demonstrated a progressive increase in network complexity from AP through AS to Tin (Figure 2). The tinnitus network featured more nodes and edges, denser local interactions, stronger modular organization, and a more centralized hub-driven architecture compared with the physiological auditory perception network. These structural properties are consistent with aberrant synaptic transmission, maladaptive plasticity, and enhanced central gain in the cingulate gyrus during tinnitus pathogenesis. Full quantitative topological metrics and description are available in Table S1 and its note.

We analyzed the frequency distributions of node degree and combined score (CS) to establish robust thresholds for identifying HDPs and HSIPs across the AP, AS, and Tin networks (Figure 3). Detailed distribution characteristics and quantitative thresholds for all three networks are provided in the legend of Figure 3. Overall, the Tin network harbored a substantially larger set of highly connected hub proteins and exhibited markedly higher connectivity and structural complexity than both the AP and AS networks.

### 2.4. Key Proteins of the PPI Networks of the AP, AS and Tin Lists

In PPI networks, HDPs and their HSIPs are recognized as functionally critical components, as they govern extensive protein connections and mediate core signaling pathways. The combined score (CS) was utilized to define HSIPs, since it integrates multiple lines of evidence, including coexpression, experimental validation, and text mining, thus providing a robust measure for identifying biologically meaningful interactions. Based on the frequency distribution of CS values, the 90th percentile was applied as the threshold to screen HSIPs (AP: > 0.934; AS: > 0.862; Tin: > 0.891). The key proteins for each network, comprising the top two HDPs and their corresponding HSIPs, are summarized in Table 4. In the AP network, CHAT (degree = 4) and TARDBP (degree = 3) were identified as the major HDPs. CHAT exhibited strong interactions with CHRNA9 and CHRNA10 as HSIPs, while TARDBP interacted with FUS and PHB2 at high confidence. In the AS network, SLC6A3 (degree = 11) and DRD1 (degree = 7) represented the top HDPs. SLC6A3 showed the highest-confidence interactions with DRD4 and PRKCA, and DRD1 interacted intensively with SLC6A2 and OPRM1. In the Tin network, *TNF* (degree = 22) and *AKT1* (degree = 21) were identified as the two most prominent HDPs. *AKT1* exhibited robust high-confidence interactions with HSP90AA1 and BCL2, both of which met the HSIP threshold (CS > 0.891). *TNF*, although the highest-degree node in the network, had no interactions that reached the strict 90th percentile CS cutoff for HSIPs; GAPDH (CS = 0.849) and BCL2 (CS = 0.885) are representative interacting partners of *TNF*. These key proteins were characterized by high closeness centrality, betweenness centrality, and edge betweenness, supporting their central roles in maintaining network stability and regulating signaling transmission in tinnitus.

To further characterize the functional roles of the identified key proteins in the cingulate gyrus, we performed Gene Ontology (GO) enrichment analysis (*p* < 0.05) of the key proteins. A total of 13, 26, and 20 GO terms were enriched in the AP, AS, and Tin groups, respectively (Table 4). For cellular components (CCs), key proteins in the AP group were strongly enriched in neuron projection, cholinergic synapse, neurotransmitter receptor complex, intracellular membraneless organelle, acetylcholine-gated channel complex, and postsynaptic specialization membrane, driven mainly by CHAT, CHRNA9, CHRNA10, FUS, and TARDBP. In the AS group, the CC terms were dominated by presynaptic membrane, plasma membrane, cell projection, neuron projection, axon, dendrite, membrane, synapse, and endoplasmic reticulum, involving DRD1, SLC6A2, SLC6A3, OPRM1, DRD4, and PRKCA. In the Tin group, CC terms were significantly enriched in protein-containing complex and myelin sheath, centered on HSP90AA1, BCL2, *AKT1*, and *TNF*.

For biological processes (BPs), AP key proteins were involved in the serotonin receptor signaling pathway, detection of mechanical stimulus involved in sensory perception of sound, response to auditory stimulus, and membrane depolarization, consistent with normal auditory processing and cholinergic synaptic transmission. In the AS group, BP terms were predominantly associated with dopamine metabolic process, G protein-coupled receptor signaling pathway, dopamine uptake, response to xenobiotic stimulus, norepinephrine uptake, dopaminergic synaptic transmission, and behavioral fear response, highlighting synaptic transmission and stress-related modulation. In the Tin group, BP terms were strongly enriched in negative regulation of apoptotic signaling pathways, positive regulation of nitric oxide biosynthetic process, glucose metabolic process, regulation of protein localization, response to xenobiotic stimulus, regulation of apoptotic process, and apoptotic mitochondrial changes, indicating prominent roles in anti-apoptosis, stress response, and metabolic regulation in tinnitus.

Together, these results demonstrate that key proteins in the AP network underlie normal auditory and cholinergic synaptic function, that those in the AS network mediate synaptic plasticity and stress-related neurotransmission, and that those in the Tin network are predominantly involved in apoptosis, stress adaptation, and survival signaling within the cingulate gyrus. Collectively, these findings provide a molecular basis for tinnitus pathogenesis.

### 2.5. Transcriptomic Validation of the AKT1 Axis and Extracellular Matrix Remodeling in the Cingulate Gyrus of Tinnitus Mice

Based on the hub gene list obtained from preceding bioinformatic screening, in vivo experiments were implemented to verify the expression change in core candidate *Akt1* and profile overall pathway alterations in the cingulate gyrus of tinnitus mice. First, longitudinal GPIAS tests were conducted to confirm reliable tinnitus-like phenotype after noise exposure (Figure 4A). Compared with baseline measurement on Day-3, statistically significant GPIAS reduction was observed on Day 1 and Day 7, while GPIAS on Day 14 recovered to the baseline level without significant difference. Cingulate gyrus tissues were harvested at Day 1 post noise exposure, a time point with markedly impaired GPIAS responses indicative of stable tinnitus-like symptoms, for subsequent transcriptomic analysis. The 100 dB SPL 2 h broadband noise exposure paradigm used here has been previously shown in C57BL/6J mice to induce primarily a temporary auditory threshold shift that fully recovers by Day 14, with no permanent hearing loss across standard test frequencies [21]. Of note, auditory brainstem response (ABR) thresholds were not assessed in this study; therefore, this behavioral deficit cannot be definitively dissociated from the acute temporary threshold shift present at this early time point. RNA-seq differential expression analysis was conducted with thresholds set as |log2FC > 1| and *p* < 0.05 (Figure 4B). A total of 116 differentially expressed genes (DEGs) were identified in the cingulate gyrus of tinnitus mice, among which 21 genes were significantly upregulated and 95 genes were significantly downregulated. Consistent with bioinformatic screening outcomes, transcriptional upregulation of *Akt1* was observed in tinnitus samples; multiple genes related to extracellular matrix function also exhibited significant expression changes. Subsequent GO enrichment analysis, performed on the full set of differentially expressed genes, demonstrated enrichment of terms related to extracellular matrix organization, cell adhesion and cytokine receptor activity (Figure 4C). This pathway enrichment reflects genome-wide transcriptomic alterations and is not restricted to genes associated with the bioinformatically identified hub proteins. KEGG classification further indicated that these genes were concentrated in signal transduction, immune system and cell interaction pathways closely associated with extracellular matrix remodeling (Figure 4D). These enrichment results suggested that transcriptional upregulation of *Akt1* and extensive extracellular matrix-related transcriptomic alterations occur concurrently in the cingulate gyrus of mice with noise-induced tinnitus-like behavior. qPCR was further applied to quantify mRNA expression of *Akt1* (Figure 4E). Significant upregulation of *Akt1* mRNA in the tinnitus group validated the RNA-seq findings, consolidating the findings that abnormal activation of *AKT1* and subsequent extracellular matrix remodeling constitute key molecular alterations of cingulate gyrus during tinnitus.

## 3. Discussion

### 3.1. Key Proteins Distinguish Physiological Auditory Processing, Acoustic Response and Pathological Tinnitus in the Cingulate Gyrus

Distinct hub protein modules governing auditory perception, acoustic stimulation and tinnitus were identified via multi-database bioinformatic screening, which resolved the molecular differentiation of cingulate gyrus under physiological, stimulated and pathological states.

For the physiological auditory perception network, CHAT and TARDBP were identified as core regulatory proteins. CHAT-mediated cholinergic synaptic transmission maintains normal sensory gating within the cingulate gyrus, supporting stable sound signal detection without excessive neural excitation. Its high-confidence interactions with nicotinic acetylcholine receptor subunits CHRNA9 and CHRNA10 sustain baseline auditory synaptic function, consistent with previously reported cholinergic modulation of cortical auditory attention. TARDBP and its binding partners FUS and PHB2 are responsible for RNA processing and mitochondrial homeostasis, serving as foundational molecules to maintain normal neuronal stability under resting auditory conditions. The relatively sparse connectivity of the AP PPI network reflects a balanced, low-excitation state of cingulate circuits during routine sound perception.

Under acute acoustic stimulation, the network shifted toward dopaminergic signaling dominated by SLC6A3 and DRD1. As key regulators of dopamine transport and postsynaptic dopaminergic response, these two proteins coordinate norepinephrine and opioid signaling cascades to trigger transient arousal and stress-related neural plasticity. The elevated network connectivity and clustering coefficient of the AS network indicate a temporary adaptive response of the limbic–auditory circuit upon external sound load, which only induces reversible synaptic adjustment without persistent pathological remodeling.

In contrast, the tinnitus network exhibited a drastically reorganized topological structure centered on *TNF* and *AKT1*, the two highest-degree hub proteins that form a putative inflammatory–survival signaling module based on PPI topological analysis. *TNF* acts as a pro-inflammatory cytokine that drives microglial activation, enhances neuronal excitability and amplifies synaptic noise within limbic regions, while *AKT1* mediates anti-apoptotic signaling and metabolic compensation to counteract chronic inflammatory stress. Unlike AP and AS networks dominated by neurotransmitter transport and transient synaptic regulation, the Tin hub proteins are mainly enriched in anti-apoptotic, nitric oxide metabolism and stress response pathways. Notably, multiple extracellular matrix regulatory genes were simultaneously enriched in the tinnitus-related gene pool, implying extracellular matrix remodeling as a parallel pathological event accompanying inflammatory–survival signaling.

Most prior bioinformatic investigations of tinnitus only mined single-source datasets and prioritized classic auditory nuclei such as the inferior colliculus and primary auditory cortex, while systematic screening of cingulate gyrus molecules and in vivo experimental validation remain scarce. The present multi-database screening strategy overcomes the bias of single-source gene-list analysis and further identifies *AKT1* and *TNF* as unique pathological hub proteins specific to tinnitus rather than general auditory stimulation.

### 3.2. Progressive Reorganization of PPI Network Topology Along the Transition from Normal Hearing to Tinnitus

Comparative topological analysis of three PPI networks revealed a progressive structural reorganization of cingulate molecular interaction networks during the progression from physiological auditory perception to tinnitus. The continuous increase in node number, edge number and average neighbor count from AP to Tin demonstrates progressive recruitment of functional proteins to support sustained pathological signal transmission.

Reduced characteristic path lengths and elevated clustering coefficient in AS and Tin networks facilitate rapid signal propagation and localized modular signal amplification, which provides a molecular basis for the central gain enhancement widely recognized in tinnitus pathogenesis. The highest network heterogeneity and centralization observed in the Tin network further confirm that pathological signaling in tinnitus relies heavily on the dominant regulatory effects of hub proteins *AKT1* and *TNF*.

This topological transformation pattern is consistent with neuroimaging observations of hyperconnectivity between auditory and limbic circuits in chronic tinnitus patients. Previous studies only described macroscopic functional connectivity abnormalities of the cingulate gyrus, whereas the present topological data offer a molecular network explanation for hyperplastic limbic–auditory coupling, which supplements the macroscopic imaging findings at the protein interaction level.

### 3.3. Independent Molecular Signatures of AKT1 Upregulation and Extracellular Matrix Remodeling Validated by In Vivo Transcriptomics

To eliminate the limitation of pure bioinformatic prediction lacking in vivo evidence, noise-induced tinnitus mouse models recapitulating clinical noise-triggered acquired tinnitus were adopted for transcriptomic verification in this study. Longitudinal GPIAS behavioral tests confirmed stable tinnitus-like auditory dysfunction at Day 1 post noise exposure, at which time cingulate gyrus tissues were collected for RNA-seq detection.

Transcriptomic profiling identified 116 DEGs in cingulate gyrus tissue from tinnitus-model mice, including 21 upregulated and 95 downregulated genes. Consistent with bioinformatic prediction, the hub gene *Akt1* displayed significant transcriptional upregulation in the tinnitus group, which was further validated by qPCR quantification. Notably, *Tnf* transcript levels did not reach statistical significance in the bulk tissue RNA-seq analysis, and the direct functional interaction between *AKT1* and *TNF* was not experimentally assessed in this study. This in vivo evidence verifies the reliability of *AKT1* as a core tinnitus biomarker screened from multi public databases, distinguishing this work from previous purely computational tinnitus research without animal validation.

GO enrichment analysis based on all DEGs revealed prominent enrichment of terms related to extracellular matrix organization, cell adhesion and cytokine receptor activity. This ECM pathway signature was derived from the full transcriptomic dataset, and a direct regulatory link between *AKT1* signaling and these ECM-related transcriptional changes have not been established in this study. Extracellular matrix components modulate synaptic stability, intercellular signal transmission and glial–neuronal crosstalk; sustained matrix remodeling may disrupt normal sensory gating and aggravate aberrant neural synchronization within limbic circuits. It is worth emphasizing that the present transcriptomic data only confirm that *AKT1* elevation and extracellular matrix pathway disturbance coexist in tinnitus lesions, and no causal regulatory relationship between the two molecular events is inferred without further functional intervention experiments.

The coexistence of inflammatory hub activation and extracellular matrix structural disorder provides a dual molecular framework to interpret why stress and inflammatory responses jointly exacerbate tinnitus distress mediated by the cingulate gyrus.

### 3.4. Cingulate Gyrus as a Core Limbic Hub Linking Tinnitus Sensation and Negative Emotional Distress

Cumulative clinical imaging evidence confirms that anterior cingulate gyrus dys-function is tightly correlated with tinnitus-induced anxiety, sleep disturbance and emotional discomfort, yet the underlying molecular cascade remains unclear. The lay-ered molecular signatures identified in this study establish a progressive pathological chain within the cingulate gyrus: physiological cholinergic transmission for sound per-ception, transient dopaminergic stress response upon acoustic stimulation, and persistent AKT1-TNF inflammation coupled with extracellular matrix remodeling under tinnitus.

This layered molecular transition explains the clinical phenomenon that hearing loss or acoustic trauma alone cannot induce chronic tinnitus, while persistent inflammatory stress and limbic microenvironment remodeling jointly drive the conversion from transient auditory discomfort to persistent distressful tinnitus. Most existing mechanistic studies separate auditory sensory signals and emotional regulation pathways; the present work integrates multi-database bioinformatics and in vivo transcriptomics to connect inflammatory–survival signaling and extracellular microenvironment changes in the cingulate gyrus, offering a novel molecular perspective for interpreting the emotional comorbidity of tinnitus.

### 3.5. Translational Prospects

The integrated multi-database bioinformatic and in vivo transcriptomic strategy adopted in this work effectively overcomes the major defect of single-source computational analysis without experimental validation in prior tinnitus research. The identified *AKT1*-centered molecular signature and extracellular matrix remodeling signature provide candidate molecular biomarkers and potential therapeutic targets for subsequent tinnitus mechanistic research and preclinical drug development. Targeted interventions against *AKT1*-mediated inflammatory signaling or extracellular matrix degradation in the cingulate gyrus may relieve both the phantom perception associated with tinnitus and accompanying emotional distress in future clinical treatment.

## 4. Materials and Methods

### 4.1. Gene List Construction

Three gene lists associated with auditory perception (AP), acoustic stimulation (AS), and tinnitus (Tin) in the cingulate gyrus (CG) were constructed using the GeneCards database (https://www.genecards.org/ accessed on 1 April 2026) with the following keyword combinations: (a) “Cingulate Gyrus” AND “synaptic transmission” AND “auditory perception”, indicative of the physiological hearing process and summarized under the term “Auditory Perception” (AP); (b) “Cingulate” AND “synaptic transmission” AND “acoustic stimulation”, indicative of activation of the auditory system (Acoustic stimulation, AS); and (c) “Cingulate Gyrus” AND “synaptic transmission” AND “tinnitus”, indicative of the process of tinnitus.

### 4.2. Characterization of the Gene Lists

The gene lists were characterized by analyses of gene overlap using Venn diagrams (http://bioinformatics.psb.ugent.be/webtools/Venn/ accessed on 1 April 2026). The Database for Annotation, Visualization, and Integrated Discovery (DAVID, https://davidbioinformatics.nih.gov/ accessed on 1 April 2026) was used for the gene-enrichment analysis and the identification of Gene Ontology (GO) terms.

### 4.3. Protein–Protein Interaction (PPI) Network Construction

PPI networks were constructed using the STRING database (https://cn.string-db.org/ accessed on 10 April 2026. Cytoscape NetworkAnalyzer (Cytoscape_3_10_4_windows_64bit, accessed on 10 April 2026) was used to identify the topological criteria and functionally important proteins in the PPI network, including the number of nodes and edges, average number of neighbors, characteristic path length, clustering coefficient, network density centralization, and heterogeneity. The node degree and the combined score (CS) for interacting proteins were used to identify the functionally important proteins. Due to potential biases inherent in protein lists and to ensure analytical focus, only the top two HDPs were retained for further investigation. The combined score (CS) characterizing interactions between HDPs and their partners integrates multiple lines of evidence, including gene coexpression, experimentally validated interactions, and automated text mining [22]. We hypothesized that HDPs and their correspondingly high-confidence interacting partners (designated high-score interaction proteins, HSIPs)—collectively termed key proteins—serve as functionally critical regulators within protein–protein interaction networks. In this study, HSIPs were defined using a stringent threshold of CS value exceeding the 90th percentile of the global CS frequency distribution.

### 4.4. Noise-Induced Tinnitus Mouse Model

Six-week-old male C57BL/6J mice were obtained from Shulaibao (Wuhan) Biotechnology Co., Ltd.y) for these experiments. All mice were raised in the specific-pathogen-free Experimental Animal Center of Huazhong University of Science and Technology. The mice were housed on a 12 h light/dark cycle with free access to food and water, and all experiments were performed during the light phase of the cycle. Noise-induced tinnitus models were selected for in vivo validation, as acoustic overstimulation represents the most prevalent clinical trigger of acquired human tinnitus and recapitulates core behavioral and molecular pathological features of noise-related tinnitus. Anesthetized C57BL/6 mice aged 6 weeks were exposed to continuous broadband white noise at 100 dB SPL for 2 h using a speaker positioned 15 cm above the mice’s heads in a sound-isolated chamber. A 24 h recovery period was arranged after noise exposure before behavioral detection and tissue sampling. Age-matched mice without noise stimulation were set as the control group. All experimental procedures were conducted in accordance with the policies of the Committee on Animal Research of Tongji Medical College, Huazhong University of Science and Technology (IACUC Number: 4256; approval date July 24, 2026).

### 4.5. GPIAS Behavioral Assessment for Tinnitus Phenotype

All behavioral assessments were performed on eight independent mice (n = 8). Each mouse served as its own control, with measurements collected at four predefined time points including pre-noise baseline, day 1, day 7 and day 14 after noise exposure. The GPIAS paradigm used to assess tinnitus-related behavioral deficits followed detailed procedures reported in prior studies [21]. GPIAS assay was performed at four time points: Day-3 (baseline before noise exposure), Day 1, Day 7 and Day 14 after noise exposure. The startle response induced by loud background noise with or without a gap prepulse was recorded and quantified. Significant decline in GPIAS value relative to baseline was defined as successful establishment of tinnitus phenotype.

### 4.6. RNA Sequencing and Differential Expression Analysis

Total RNA was extracted from cingulate gyrus tissues of tinnitus and control mice. RNA quality inspection, library construction and transcriptome sequencing were completed on the Illumina sequencing platform. Raw sequencing data were quality-controlled and mapped to the mouse reference genome. Differentially expressed genes (DEGs) were screened with thresholds of |log2FC > 1| and *p* < 0.05. Volcano plot was generated to visualize overall transcriptional changes between groups. GO and KEGG enrichment analyses were conducted to interpret functional characteristics of all DEGs.

### 4.7. Quantitative Real-Time PCR (qPCR) Verification

The mice were deeply anesthetized and subsequently euthanized by cervical dislocation. Six biological replicates (n = 6) were included in the tinnitus group and six biological replicates (n = 6) in the control group. Total RNA reverse transcription was conducted to synthesize complementary DNA (cDNA) [23,24,25,26,27]. Primer sequences specific to *Akt1* and internal reference gene were designed for qPCR amplification. The relative mRNA expression level of *Akt1* was calculated via the 2^−ΔΔCt^ method, and expression differences between tinnitus and control groups were statistically compared.

### 4.8. RNA Isolation, Library Construction and Sequencing

Total RNA was extracted from cingulate gyrus tissue using the Total RNA Extractor (Trizol) kit (B511311, Sangon, Shanghai, China) following the manufacturer’s instructions, and treated with RNase-free DNase I to eliminate genomic DNA contamination [28,29,30,31]. RNA integrity was verified by 1.0% agarose gel electrophoresis. RNA purity and concentration were measured using a NanoPhotometer spectrophotometer (IMPLEN, Westlake Village, CA, USA) and Qubit 2.0 Fluorometer (Invitrogen, Carlsbad, CA, USA). For RNA-seq, four biological replicates were included for the control group and four biological replicates for the tinnitus group; each biological replicate represented cingulate gyrus tissue collected from an individual mouse. Qualified RNA samples were delivered to Sangon Biotech (Shanghai, China) Co., Ltd. for library preparation and transcriptome sequencing.

Briefly, mRNA was enriched from total RNA by oligo-dT magnetic beads. The purified mRNA was fragmented into short fragments using divalent cations under high temperature. Double-stranded cDNA was synthesized with fragmented mRNA as the template. After end repair, dA-tailing and sequencing adapter ligation, suitable-size cDNA fragments were purified, circularized to form DNA nanoballs (DNBs), and amplified by rolling-circle replication. Sequencing was performed on the DNBSEQ-T7 platform (MGI, Shenzhen, China) with paired-end 150 bp (PE150) strategy. Approximately 6 Gb clean sequencing data were targeted for each sample.

### 4.9. Data Assessment and Bioinformatic Analysis

FastQC (version 0.11.2) was applied to evaluate raw read quality. Raw reads were filtered by Trimmomatic (version 0.36) to remove adapter sequences, low-quality bases and short reads shorter than 35 nt; the resulting clean reads were used for downstream analysis. Clean reads were mapped to the mouse reference genome using HISAT2 (version 2.0) with default parameters. Transcript-level gene expression was quantified by StringTie (version 1.3.3b), and TPM values were calculated for gene expression comparison across samples. Differentially expressed genes (DEGs) between tinnitus and control groups were identified by DESeq2 (version 1.12.4). Genes with *p*-value ≤ 0.001 and |log2FC > 1| were regarded as significantly differentially expressed. Functional enrichment analyses of Gene Ontology (GO) and KEGG pathways were performed for DEGs; terms with false discovery rate q-value < 0.05 were considered significantly enriched [32,33].

## 5. Conclusions

The PPI networks and key proteins in the CG differ significantly between auditory perception, acoustic stimulation, and tinnitus. Tinnitus is characterized by hyperconnected, centralized networks with *AKT1* and *TNF* as top-ranked hub nodes, which are predicted to be involved in inflammatory signaling, abnormal excitability, and maladaptive plasticity. These findings provide novel candidate targets and mechanistic insights for understanding the molecular basis of tinnitus-related distress in the cingulate gyrus.

### Limitations

Several limitations of this study should be noted. First, the noise exposure paradigm used here is a well-established and clinically relevant model for inducing tinnitus-like phenotypes in rodents, and all animals in the tinnitus group were confirmed positive for tinnitus-like behavior via GPIAS testing. However, with this experimental design, the molecular alterations observed cannot be fully disentangled from the general effects of acoustic injury and stress responses triggered by noise exposure itself. This is an inherent caveat shared by nearly all externally induced animal models of tinnitus, including salicylate administration and ototoxic drug-induced models, because the pathological phenotype is secondary to the inducing insult, and no ideal control group that develops tinnitus entirely independently of the triggering injury can be practically established in vivo. Accordingly, the findings of this study are interpreted as molecular correlates of a noise-induced tinnitus-like phenotype, rather than as tinnitus-specific biomarkers. Second, tissues were collected at Day 1 post noise exposure, which reflects the acute onset phase of tinnitus; whether these molecular changes persist into the chronic phase remains to be determined. Future studies incorporating noise-exposed animals without tinnitus-like behavior as an additional control group, and analyses across multiple time points, will help to further dissociate general acoustic injury effects from tinnitus-specific molecular signatures. Third, ABR thresholds were not measured alongside GPIAS testing. Even so, the observed reduction in GPIAS at Day 1 and Day 7 may reflect a combination of tinnitus-like perceptual disturbance and temporary acoustic threshold shift, and cannot be exclusively attributed to tinnitus. This represents an important confounding variable; future studies combining serial ABR measurements with GPIAS behavioral testing will be needed to dissociate the contributions of hearing threshold shift and tinnitus to the molecular alterations observed in the cingulate gyrus. Fourth, the initial gene lists were retrieved from the GeneCards database via keyword-based search, which inevitably includes a broad set of genes associated with general synaptic transmission as a shared molecular background across all three groups. To mitigate this bias, we applied subsequent PPI topological analysis with stringent degree and combined score cutoffs to prioritize functionally critical hub molecules for downstream interpretation.

## Figures and Tables

**Figure 1:**
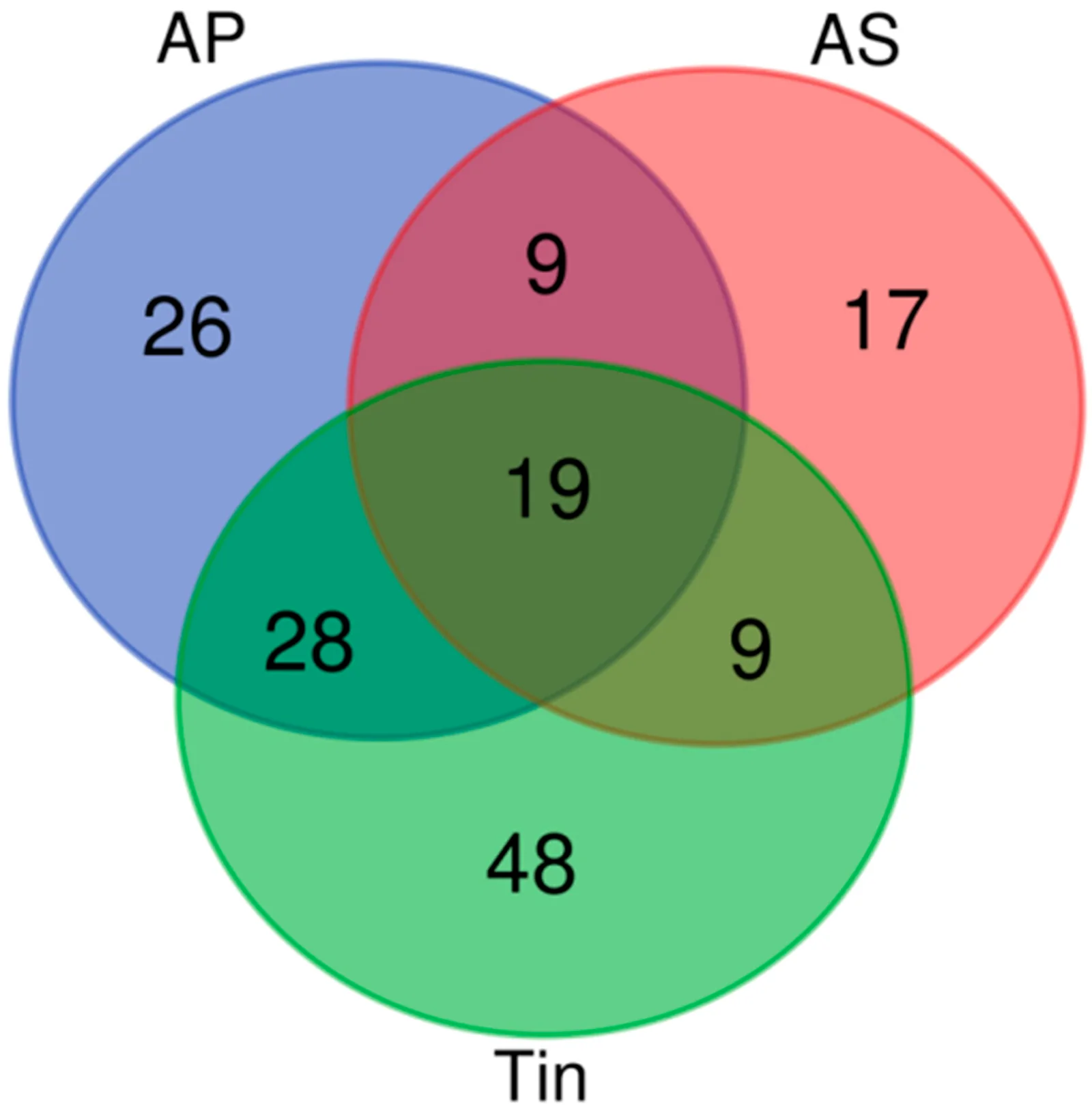
Venn diagram of the genes involved in AP, AS and Tin processes.

**Figure 2:**
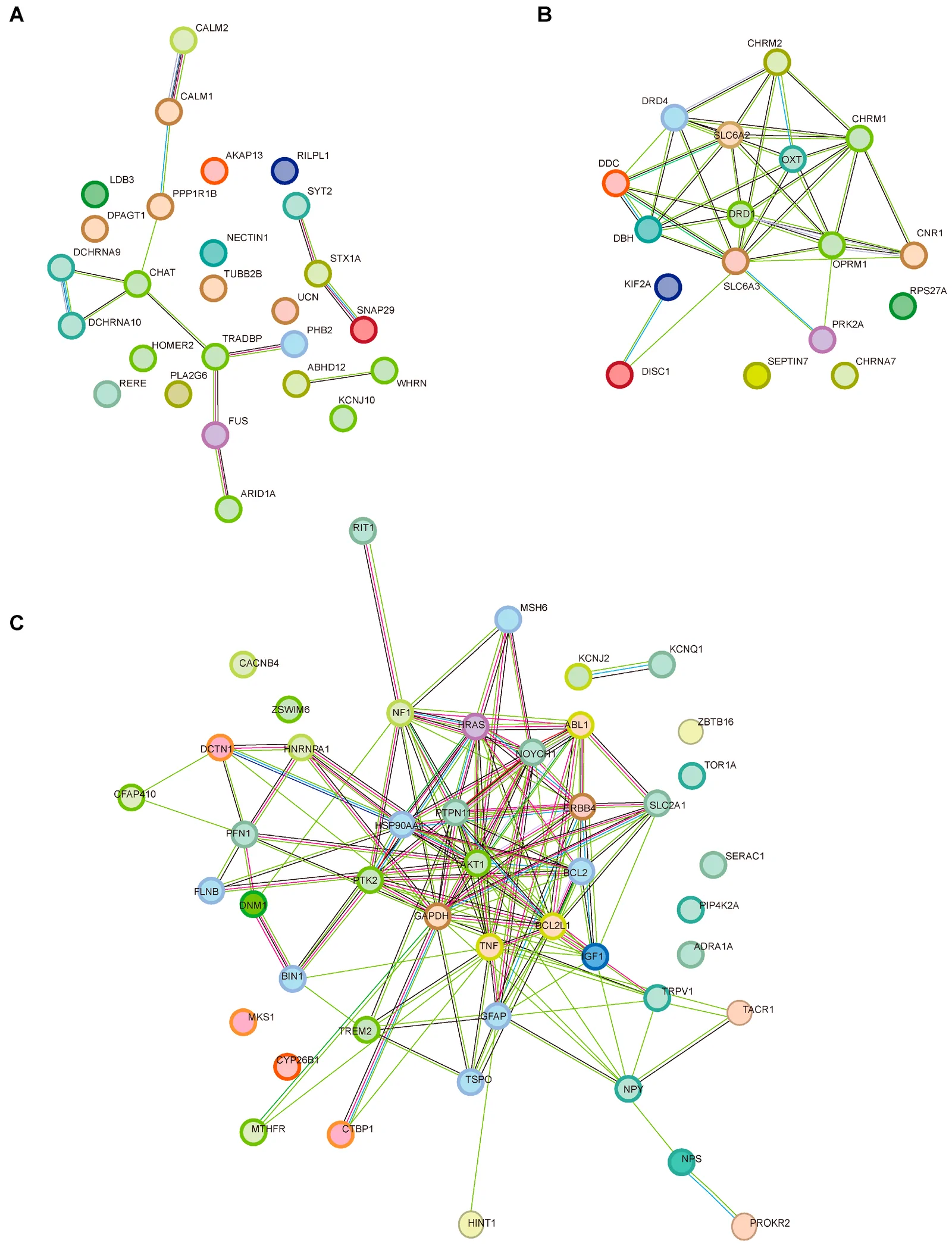
PPI network of the AP, AS, and Tin processes in the cingulate gyrus. (**A**) PPI network of the AP process. (**B**) PPI network of the AS process. (**C**) PPI network of the Tin process.

**Figure 3:**
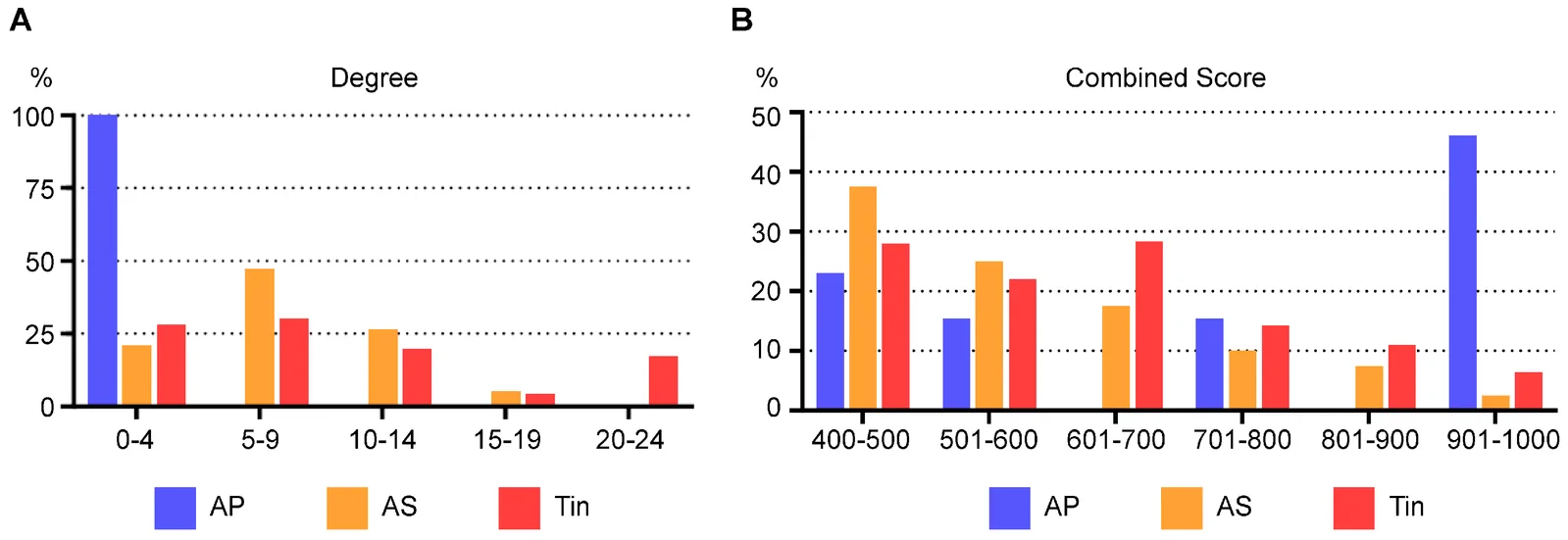
Frequency distribution analyses of node degree and combined score (CS) values in the AP, AS, and Tin PPI networks. (**A**) Percentage frequency distribution of node degree. All three networks exhibited a bell-shaped distribution, a typical feature of scale-free biological networks. In the AP network, all nodes had a degree between 0 and 4, consistent with a loosely connected modular architecture. In the AS network, 47.40% of nodes fell into the 5–9 degree interval, followed by the 0–4 (21.10%) and 10–14 (26.30%) intervals. The Tin network showed a much wider degree distribution: 28.30% of nodes in the 0–4 range, 30.40% in the 5–9 range, and 17.40% in the 20–25 range, forming a distinct cluster of highly connected hub proteins. Characteristic values of the degree frequency curves (AP/AS/Tin): median = 2/6/8; 90th percentile = 4/11/21. (**B**) Percentage frequency distribution of CS values. The frequency of interactions decreased gradually with increasing CS values in all three groups. In the AP group, 46.15% of interactions were concentrated in the 901–1000 interval, representing the highest proportion of high-confidence interactions. In the AS network, 80% of all edges fell into the low-to-moderate CS range of 400–700, with only 2.5% reaching scores above 900. In the Tin network, CS values spread more broadly across the 400–800 interval, with 6.49% of interactions landing in the 901–1000 bracket. Characteristic values of the CS frequency distribution (AP/AS/Tin): median = 495/585/652; 90th percentile = 934/862/891. The 90th percentile of the CS distribution was used as the cutoff to define HSIPs. The frequency distributions of degree and CS values were calculated as percentages of the total number of nodes and edges, respectively. Degree values are binned at intervals of 5; CS values are binned at intervals of 100, where class 1 corresponds to CS = 400–500 and class 6 corresponds to CS = 901–1000.

**Figure 4:**
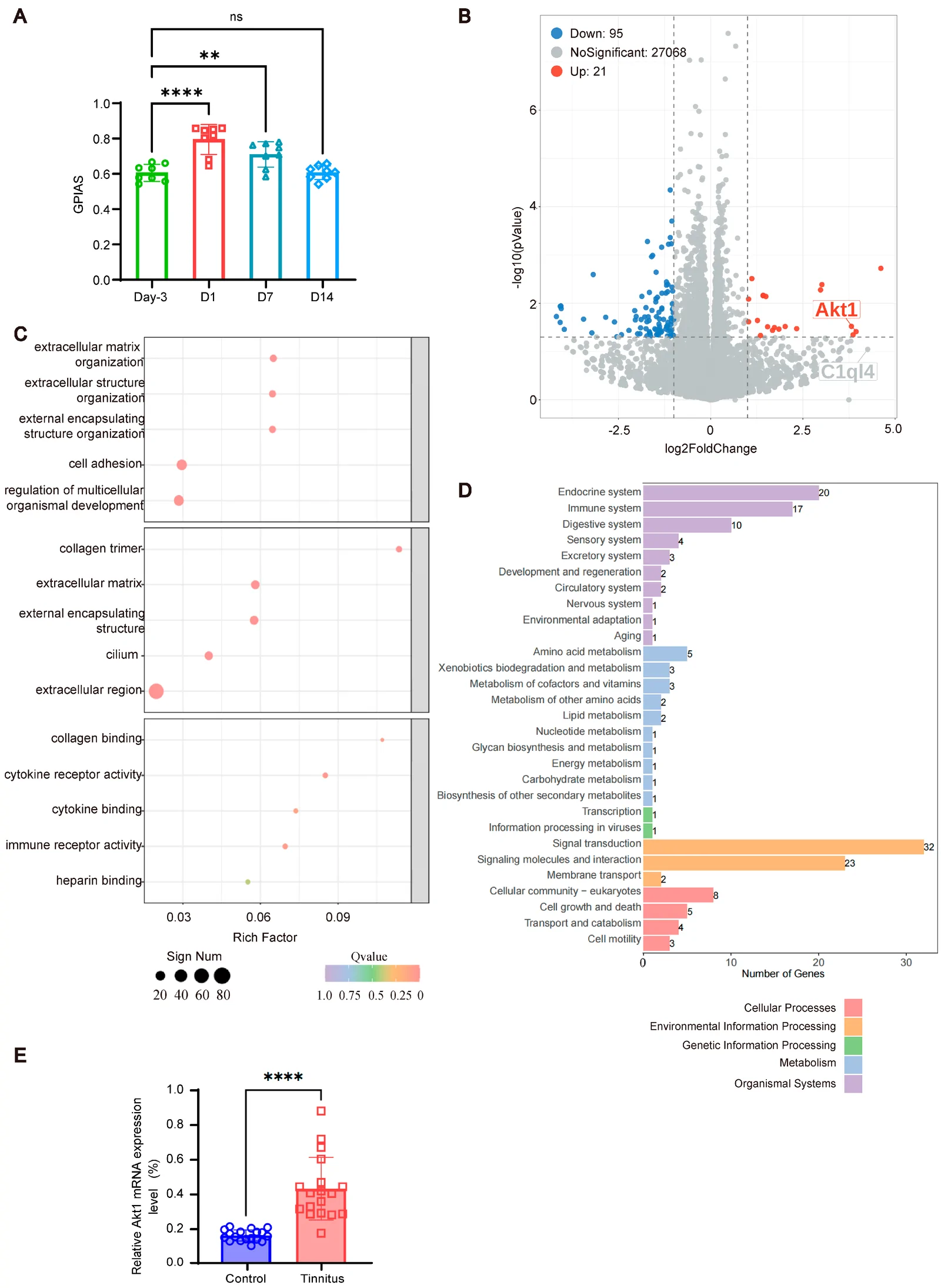
Transcriptomic and phenotypic validation of bioinformatically predicted hub gene *Akt1* and extracellular matrix pathway alterations in the cingulate gyrus of noise-induced tinnitus mice. (**A**) Longitudinal GPIAS test for the verification of tinnitus-like behavioral phenotype after noise exposure (n = 8). (**B**) Volcano plot of differentially expressed genes identified by RNA-seq (n = 4) in cingulate gyrus tissues from tinnitus and control mice. (**C**) Top enriched GO terms of differentially expressed genes. (**D**) KEGG pathway classification of differentially expressed genes. (**E**) qPCR quantification of *Akt1* mRNA expression in noise-induced tinnitus mice cingulate gyrus. ** *p* < 0.01, and **** *p* <0.0001.

**Table 1:** Distribution of genes of the three gene lists among the different groups of the Venn diagram.

Process	Number	Genes
AP/Acoustic/Tin	19	*CACNA1A BDNF TH PSEN1 CDH23 SLC6A4 NCAM1 ATP1A2 KIF1B BDNF-AS HRH1 MAPT APP COMT DNMT3A SOD1 APOE POMC NR3C1*
AP/Acoustic	9	*HTR2A PLP1 HTR1A PVALB DRD2 HTR3A NTRK2 SLC6A1 MYO15A*
AP/Tin	28	*CACNA1C MAP1B LMNA SLC1A3 SNAP25 PMP22 TRPV4 RNF220 DOCK4 NIPBL STIM1 POLG GNRH1 AGO2 RYR1 MBP HDAC4 NTF3 PIK3R1 GSK3B PDYN CHD7 CAMK2G SOX2 LRP4 USP48 VAMP1 S100B*
Acoustic/Tin	9	*LOC110806262 GRIN1 GABBR1 SNCA RELN TCF4 SLC12A5 SYP CREB1*
AP	26	*SNAP29 PPP1R1B PLA2G6 KCNJ10 LDB3 CHRNA10 NECTIN1 PHB2 RILPL1 FUS AKAP13 UCN DPAGT1 TUBB2B CALM1 STX1A ABHD12 CALM2 WHRN CHAT HOMER2 ARID1A TARDBP SYT2 CHRNA9 RERE*
Acoustic	17	*DISC1 SLC6A2 SEPTIN7 CRHR1 RPS27A OXT DBH DRD4 KIF2A PRKCA DDC SLC6A3 CNR1 CHRM2 CHRNA7 DRD1 OPRM1*
Tin	48	*CYP26B1 ERBB4 KCNJ2 PTK2 DNM1 ZSWIM6 NPS BCL2 IGF1 HRAS MKS1 GAPDH CTBP2 DCTN1 TNF PROKR2 MIR9-1 BCL2L1 TACR1 ABL1 ZBTB16 CACNB4 HINT1 AKT1 NOTCH1 HNRNPA1 MTHFR PTPN11 NF1 CFAP410 TREM2 SOX2-OT ADRA1A SERAC1 SLC2A1 PFN1 KCNQ1 RIT1 PIP4K2A TOR1A TRPV1 NPY HSP90AA1 FLNB BIN1 TSPO MSH6 GFAP*

**Table 2:** Top five GO terms for cellular components and biological processes in the cingulate gyrus (CG) for the AP, AS, and Tin processes (*p* < 0.01, count = 2).

AP (25 Chart Records)	AS (25 Chart Records)	Tin (125 Chart Records)
Cellular components: *p* = 1.31 × 10^−4^ to 6.72 × 10^−4^ Fold enrichment: 6.4–85.75	Cellular components: *p* = 1.95 × 10^−9^ to 3.01 × 10^−3^ Fold enrichment: 9.22–12.77	Cellular components: *p* = 9.47 × 10^−11^ to 1.51 × 10^−5^ Fold enrichment: 1.88–8.53
Synapse (8) Schaffer collateral—CA1 synapse (4) Presynaptic cytosol (3) Synaptic vesicle membrane (4) Postsynaptic cytosol (3)	Synapse (11) Axon (5) Dendrite (5) Glutamatergic synapse (5) Ciliary basal body (4)	Glutamatergic synapse (17) Cytosol (44) Neuronal cell body (11) Synapse (16) Plasma membrane (38)
Biological processes: *p* = 3.24 × 10^−6^ to 1.25 × 10^−3^ Fold enrichment: 54.91–130.52	Biological processes: *p* = 7.88 × 10^−8^ to 2.24 × 10^−5^ Fold enrichment: 25.46–410.78	Biological processes: *p* = 5.86 × 10^−10^ to 1.70 × 10^−6^ Fold enrichment: 7.87–157.04
Response to auditory stimulus (4) Autophagosome membrane docking (3) Detection of mechanical stimulus involved in sensory perception of sound (3) Response to amphetamine (3) Regulation of synaptic vesicle exocytosis (3)	Behavioral response to ethanol (4) G protein-coupled receptor signaling pathway, coupled to cyclic nucleotide second messenger (5) Chemical synaptic transmission (6) Response to amphetamine (4) Fear response (3)	Negative regulation of cell population proliferation (15) Positive regulation of ERK1 and ERK2 cascade (11) Regulation of gene expression (12) Positive regulation of action potential (4) Intrinsic apoptotic signaling pathway in response to DNA damage (6)

In parentheses: Numbers of genes per GO term. The GO terms are ordered according to the *p*-values.

## Data Availability

The original contributions presented in this study are included in the article. Further inquiries can be directed to the corresponding author.

## Supplementary Materials

The following supporting information can be downloaded at: https://www.mdpi.com/article/10.3390/ijms27198543/s1.
