# Supplementary material for: Combined Bioinformatic Analysis and RNA Sequencing Uncover Key Tinnitus-Related Molecules in the Cingulate Gyrus
Source: Int J Mol Sci. 2026 Sep 24;27(19):8543. doi: 10.3390/ijms27198543 (PMC13665349; doi:10.3390/ijms27198543)
Supplement: Supplementary file 1 [file ijms-27-08543-s001.zip › ijms-4512879-supplementary.pdf]

**Supplementary Materials**

**Table S1.** Topological criteria of the Cingulate Gyrus networks.

| | AP | AS | Tin |
|---|---|---|---|
| Number of nodes | 15 | 14 | 37 |
| Number of edges | 13 | 39 | 152 |
| Average number of neighbors | 2.000 | 5.571 | 8.629 |
| Characteristic path length | 2.689 | 1.714 | 2.050 |
| Network diameter | 6 | 4 | 5 |
| Network radius | 3 | 2 | 3 |
| Network heterogeneity | 0.447 | 0.464 | 0.713 |
| Network centralization | 0.278 | 0.487 | 0.417 |
| Network density | 0.222 | 0.429 | 0.254 |
| Clustering coefficient | 0.217 | 0.588 | 0.563 |

***Note:*** The number of nodes and edges (Table 3) increased markedly from AP (nodes=15, edg-es=13) to AS (nodes =14, edges=39) and further to Tin (nodes=37, edges=152), indicat-ing a progressive recruitment and extensive integration of interacting proteins during the transition from normal hearing to acoustic activation and finally to tinnitus. Along the same line, the average number of neighbors climbed progressively from the AP to the Tin network, with values of 2.000, 5.571, and 8.629, respectively—a clear sign of increasingly dense local interactions. In terms of signal propagation, the characteristic path lengths for the AS (1.714) and Tin (2.050) networks were both shorter than that of the AP network (2.689), implying that information transfer became more efficient once the system was stimulated or pathologically altered. The clustering coefficient also rose markedly, from 0.217 in the AP group to 0.588 in the AS group and 0.563 in the Tin group, pointing to enhanced local clustering and modular organization in both the acoustic response and tinnitus states. Network density followed a comparable trajec-tory (AP: 0.222, AS: 0.429, Tin: 0.254), reinforcing the view that interactions were tighter and more extensive in the AS and Tin networks relative to the AP network. Notably, network heterogeneity—which reflects unevenness in degree distribu-tion—was highest in the Tin group (0.713), followed by the AS (0.464) and AP (0.447) groups, suggesting that the tinnitus network relied more heavily on a few dominant hubs. Similarly, network centralization rose in the AS (0.487) and Tin (0.417) networks compared with the AP network (0.278), consistent with the emergence of core hub proteins that steer global network architecture.
